# Revisiting the structures and phase transitions of PrNiO_3_ nickelate using symmetry-mode analysis

**DOI:** 10.1107/S1600576725009380

**Published:** 2025-11-26

**Authors:** Wajdi Cherif, José Antonio Alonso, João Elias F. S. Rodrigues

**Affiliations:** a Instituto de Ciencia de Materiales de Madrid, CSIC, Cantoblanco, Madrid, 28049, Spain; b Laboratory of Electromechanical Systems (LASEM), National Engineering School of Sfax, BPW 3038, Sfax, Tunisia; c European Synchrotron Radiation Facility (ESRF) 71 Avenue des Martyrs Grenoble 38000 France; Goa University, India

**Keywords:** *R*NiO_3_ perovskites, phase transitions, symmetry-mode analysis, metal–insulator transitions

## Abstract

We have investigated the complete sequence of temperature-induced structural phase transitions in PrNiO_3_ using high-angular-resolution synchrotron X-ray diffraction and symmetry-adapted distortion-mode analysis. By employing a high-purity sample synthesized under high pressure (3.5 GPa at 1073 K), we resolve the monoclinic (*P*2_1_/*n*) → orthorhombic (*Pbnm*) → rhombohedral (*R*3*c*) evolution over an extended temperature range (10–900 K). Beyond confirming the role of oxygen stretching modes in the insulator–metal transition at ∼130 K, we show that these phonon modes also govern the high-temperature metallic–metallic transition at ∼750 K. This work provides a comprehensive mode-resolved picture of both insulator and metallic regimes in PrNiO_3_ and highlights the central role of phonon-mediated processes in driving charge disproportionation and lattice symmetry breaking across all the crystalline phases.

## Abstract 2

A full symmetry-mode decomposition analysis of PrNiO_3_ across its temperature-induced structural phase transitions allows identification of oxygen stretching and Jahn–Teller-like modes as the primary drivers of charge disproportionation at ∼130 K, concomitant with the metal–insulator transition. These distortions persist and evolve through the high-temperature orthorhombic to rhombohedral transition.

## 1. Introduction

Nickelates with the formula *R*NiO_3_ (*R* represents a rare earth element, such as La, Pr, Nd, Sm *etc.*) are complex oxide materials that have attracted increasing interest in solid-state physics and materials science research (Medarde, 1997 ▸; Tomioka *et al.*, 2021 ▸; Catalano *et al.*, 2018 ▸; Jaramillo *et al.*, 2014 ▸; Gawryluk *et al.*, 2019 ▸; Klein *et al.*, 2021 ▸; Catalan, 2008 ▸; Wang *et al.*, 2019 ▸). These compounds belong to the perovskite family, characterized by a unique crystal structure that provides exceptional electronic, magnetic and optical properties. The rare earth nickelate perovskites exhibit a variety of intriguing phenomena, such as metal–insulator transitions at characteristic temperatures *T*_IM_, unusual magnetic properties, and strong coupling between structural, electronic and spin degrees of freedom (Medarde *et al.*, 1997 ▸; Rodrigues *et al.*, 2023 ▸, 2024 ▸; Alonso *et al.*, 2000 ▸). These characteristics make *R*NiO_3_ promising candidates for applications in advanced electronic devices such as catalysts, sensors, actuators, resistive memories and energy conversion devices (Xin *et al.*, 2020 ▸; Pandiyan *et al.*, 2023 ▸; Retuerto *et al.*, 2017 ▸; Junita *et al.*, 2022 ▸). The ability to tune their properties by varying the ionic radius of the rare earth element, applying external stresses or introducing additional doping provides a wide range of possibilities for engineering new functionalities in multifunctional materials.

Rare earth nickelates *R*NiO_3_ are electron-correlated materials, where the interplay between charge and spin order leads to a rich phase diagram, passing through antiferromagnetic insulators to paramagnetic metals. As initially described by Demazeau *et al.* (1971 ▸), these materials enjoyed a renaissance of interest after the discovery of a thermally driven insulator–metal (IM) transition depending on the rare earth ionic size. In the case of small-sized rare earths (*R* = Ho–Lu, Y), it was demonstrated that the symmetry of the lattice varies from orthorhombic (*Pbnm*) to monoclinic (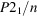) at *T*_IM_ (Fernández-Díaz *et al.*, 2001 ▸; Kobayashi *et al.*, 2015 ▸; Massa *et al.*, 2015 ▸; Alonso *et al.*, 2000 ▸; Muñoz *et al.*, 2009 ▸) to produce two chemically and crystallographically distinct Ni sites in the insulating regime, whereas the parent compound LaNiO_3_ is metallic and rhombohedral over its entire temperature range. For Pr and Nd, the IM transition coincides with the antiferromagnetic ordering temperature 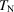, whereas for the medium-sized rare earths (*R* = Sm–Dy) 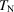 and 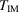 are different.

Among all the nickelates *R*NiO_3_, PrNiO_3_ occupies a unique position. It hosts all three archetypal perovskite structures (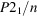, *Pbnm* and 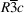) accessible via temperature tuning. Remarkably, PrNiO_3_ exhibits a simultaneous insulator–metal and antiferromagnetic transition at ∼130 K (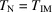), coinciding with a monoclinic to orthorhombic symmetry change, and an unusual increase in unit-cell volume upon cooling. Such a transition is believed to be stabilized by charge disproportionation, being a phonon-mediated mechanism that could potentially suppress Jahn–Teller effects. However, the interplay between lattice dynamics and electronic ordering remains poorly understood. The temperature-induced *Pbnm* to 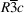 phase transition also remains largely uncharacterized, occurring in the metallic state of PrNiO_3_ above 750 K (Rodrigues *et al.*, 2024 ▸).

The phase transition 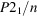 to *Pbnm*, which is simultaneously observed upon the metallization of Ni perovskites upon warming, has been analyzed in terms of symmetry-adapted distortion modes (Gawryluk *et al.*, 2019 ▸). This analysis allowed identification of the contribution of the different symmetry modes to the global distortion over a broad temperature range (Perez-Mato *et al.*, 2010*a* ▸). Moreover, it showed that the structural changes at the metal-to-insulator (MI) transition, traditionally described in terms of the evolution of the interatomic distances and angles, appear as abrupt increases of all non-zero mode amplitudes at 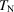
 = 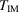
 ≃ 130 K, accompanied by the appearance of new modes below this temperature. These observations suggest the existence of a hidden symmetry in the insulating phase, which might be due to the theoretically predicted existence of polar distortions induced by the non-centrosymmetric magnetic order (Giovannetti *et al.*, 2009 ▸; Perez-Mato *et al.*, 2016 ▸). However, the underlying mechanisms of the *Pbnm* to 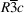 transition have not previously been analyzed using symmetry-adapted distortion modes. This is partly since the high-quality structural data necessary for this analysis have only recently become available for PrNiO_3_ through the recent work of our group (Rodrigues *et al.*, 2024 ▸).

In this paper, we revisit the evolution of distortion modes across the structural phase transitions of PrNiO_3_ nickelate, mainly addressing the metal–metal transition (*Pbnm* to 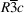). A ceramic sample was prepared at 3.5 GPa under *in situ* O_2_ pressure provided by the decomposition of KClO_3_, yielding a material with a crystallinity which exceeds those currently prepared at 200 bar O_2_ pressure (Gainza *et al.*, 2021 ▸). The transition sequence monoclinic (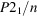) → orthorhombic (*Pbnm*) → rhombohedral (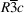) was identified in our specimen, examined with high-resolution synchrotron X-ray diffraction data (SXRD) and symmetry-adapted distortion-mode analysis.

## 2. Methods and notation

### 2.1. Experimental techniques

High-quality PrNiO_3_ powder was synthesized via high-pressure solid-state reaction (Gainza *et al.*, 2021 ▸). Stoichiometric amounts of Pr_6_O_11_ and Ni(OH)_2_ were mixed with 30 wt% KClO_4_, which generates *in situ* oxygen to oxidize Ni to the 3+ state. The mixture was sealed in a 5 mm diameter gold capsule, placed in a graphite heater and treated in a Rockland Research piston-cylinder press at 3.5 GPa and 1073 K for 30 min, and then quenched to room temperature. Residual KCl and unreacted oxides were removed by washing in dilute HNO_3_.

High-angular-resolution temperature-dependent SXRD measurements (λ = 0.35418 Å) were carried out on beamline ID22 (Fitch *et al.*, 2023 ▸) at the ESRF using a Dectris EIGER2 X 2M-W CdTe detector in position-sensitive mode, covering a 2θ range of 1–40° with an angular resolution of 0.002°. PrNiO_3_ powder was placed into a 0.5 mm diameter quartz capillary and continuously rotated to minimize orientation effects. The temperature dataset was collected in the range between 10 and 900 K with 10 min equilibration per point (Rodrigues *et al.*, 2024 ▸) using a Dynaflow cryostat (*T* < 300 K) and hot blower system (*T* > 300 K). The setup enabled precise tracking of diffraction peak splitting and distortion linked to structural transitions.

The SXRD data were analyzed by Rietveld refinement using the *FullProf* program (Rodríguez-Carvajal, 1993 ▸), with a pseudo-Voigt function employed to model the peak profiles. The refined parameters included scale factors, zero-point offset, background coefficients, asymmetry corrections, lattice constants, atomic positions, site occupancies and isotropic displacement parameters.

### 2.2. Notation

Understanding a material’s properties often relies on its electronic and phonon band structures, which are mapped within the first Brillouin zone (BZ). For a simple cubic lattice, key high-symmetry points within this reciprocal-space unit cell are crucial, namely 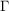, *X*, *M* and *R*. The 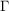 point sits at the BZ center (0, 0, 0). The *X* point at (½, 0, 0) denotes the center of a BZ face. The *M* point at (½, ½, 0) is found at the center of a BZ edge. The *R* point at (½, ½, ½) defines a BZ corner.

In Glazer’s notation, a general symbol (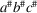) represents the tilt magnitudes along the pseudo-cubic directions *x*, *y* and *z*, respectively. The superscript # may have three values to describe no tilt (#, 0) and in-phase (#, +) and out-of-phase (#, −) octahedral tilting of the neighboring octahedral layers (Glazer, 1972 ▸, 1975 ▸). For instance, the aristotype unit cell has the Glazer symbol (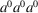), while (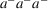) denotes out-of-phase tilts of equal magnitude along all the pseudo-cubic axes.

## 3. Structural transitions in PrNiO_3_

### 3.1. Summary of structural analysis

PrNiO_3_ undergoes a sequence of structural transitions driven by temperature and pressure. Below ∼130 K it adopts a monoclinic 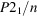 structure with charge disproportionation and antiferromagnetic order. As the temperature increases, it transitions to an orthorhombic *Pbnm* phase around 130 K, coinciding with a metal–insulator transition and the collapse of Ni site splitting. Above ∼700 K, PrNiO_3_ enters a rhombohedral 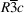 phase, associated with increased orbital overlap and metallic behavior. Similar transitions occur under pressure, with orthorhombic–rhombohedral coexistence between 6 and 12 GPa (Rodrigues *et al.*, 2024 ▸; Zhou *et al.*, 2004 ▸). Table 1 ▸ details the experimental conditions and reported transition temperatures for PrNiO_3_.

**Figure 1:**
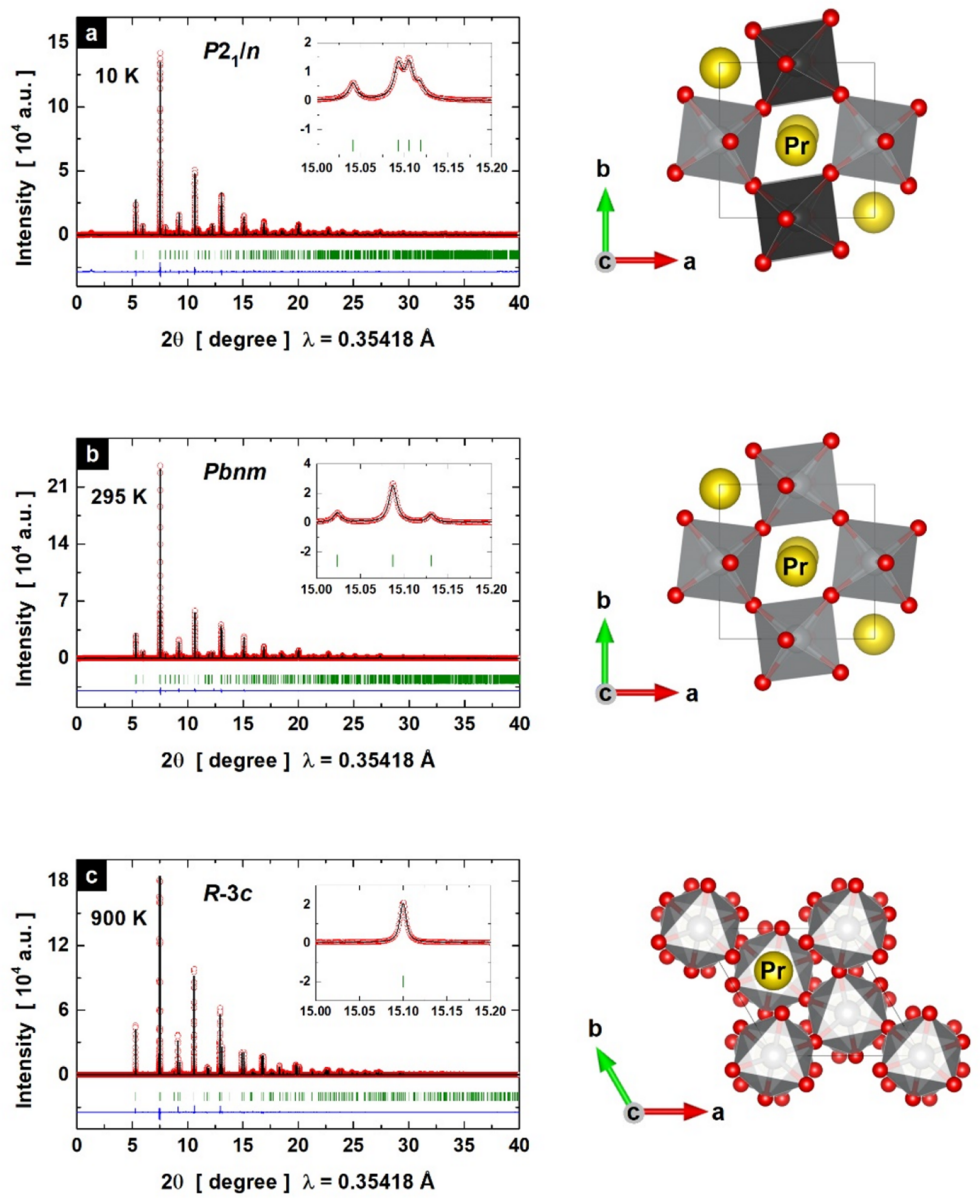
Typical high-angular-resolution SXRD profiles for PrNiO_3_ (red open circles) and corresponding Rietveld refinements (black lines) at (*a*) 10 K for the 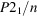 (monoclinic) phase, (*b*) 295 K for the 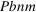 (orthorhombic) phase and (*c*) 900 K for the 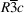 (rhombohedral) phase. Blue lines denote the fit residuals and green bars represent the expected Bragg reflections. Diagrams on the right are structural views from each phase (monoclinic, orthorhombic and rhombohedral).

In Fig. 1 ▸, we present representative SXRD patterns and corresponding Rietveld refinements of the three mentioned PrNiO_3_ structures that occur in the temperature interval of 10–900 K at ambient pressure. A characteristic feature of the monoclinic phase is the diffraction peak doublet (224 and 224 reflections) at around 15.1° (2θ) [inset of Fig. 1 ▸(*a*)]. The occurrence of this doublet is linked to the charge disproportionation on Ni sites, and thus to the generation of two distinct crystallographic Ni sites with different charges (Ni1^3+δ^ and Ni2^3−δ^). This doublet is not observed in the orthorhombic phase or for temperatures above 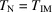 [inset of Fig. 1 ▸(*b*)]. In the orthorhombic lattice, the charge disproportionation on the Ni site collapses, resulting in a single Ni site with a uniform valence of 3+. Above 700 K, we found that a high-symmetry phase with a rhombohedral lattice is stabilized, as attested by the disappearance of the orthorhombic characteristic peaks [Fig. 1 ▸(*c*)]. All the refined parameters for the three phases are listed in Tables S1–S3 in the supporting information.

### 3.2. Distortion-mode analysis across the phase transitions

To better understand the mechanism of the phase transitions in PrNiO_3_, we used symmetry-mode analysis (also known as distortion-mode analysis). We computed the amplitudes and polarization vectors of the symmetry-adapted modes for the low-symmetry distorted phase (subgroup *H*) with respect to the high-symmetry phase (aristotype, supergroup *G*) using the *Amplimodes* algorithm available at the Bilbao Crystallographic Server (Perez-Mato *et al.*, 2010*a* ▸; Aroyo *et al.*, 2006 ▸; Perez-Mato *et al.*, 2010*b* ▸). Because the structural transitions in PrNiO_3_ exhibit group–subgroup relations between low-symmetry (*i.e.*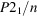, *Pbnm* and 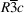) and high-symmetry structures, the low-symmetry distorted unit cell can be written as ‘frozen’ modes using irreducible representations (here referred to as irreps) of the high-symmetry space group. The high-symmetry unit cell can be hypothetical (or virtual). Here, we choose the aristotype space group (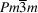), which does not present any structural distortion, such as octahedral rotation, cationic displacement or orbital ordering (Glazer, 1972 ▸). The initial point of the analysis consists of writing the atomic positions of the low-symmetry phases (**r**; subgroup *H*) with respect to the atomic positions of the aristotype (**r**_0_; supergroup *G*), being the last ones described in the unit-cell basis of the subgroup *H*, as follows:
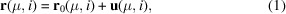
where 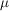 stands for distinct elements within the asymmetric unit and *i* ranges from 1 to 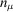 (number of atoms). For the description of the structural evolution, the displacement vectors 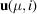 are linearly decomposed in terms of the basis vectors of the irreps obtained from the group–subgroup analysis as follows:
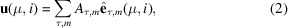
where 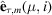 denotes the polarization vector (or basis vector) of the irrep 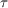 and *m* the possible independent modes for each 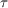, while the amplitude 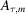 denotes the distortion magnitude of each distortion mode. A rough estimation of the uncertainty in the distortion amplitudes 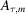, considering typical errors of ∼10^−5^ in both atomic positions (*x*, *y*, *z*) and lattice parameters (*a*, *b*, *c*), gives 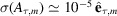, *i.e.* essentially of the order of ∼10^−5^ per unit-norm mode vector, with an increase of only a few percent from the lattice parameter contributions.

To perform the symmetry-mode decomposition, we used the experimentally determined structures defined in 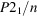, *Pbnm* and 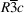 space groups, as reported by Rodrigues *et al.* (2024 ▸), constituting the subgroup *H*, while the high-symmetry supergroup *G* contains the unit cell 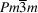. For this high symmetry, we took the virtual aristotype cubic structure belonging to space group 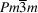 (No. 221), where Pr is located on 1*b* (½, ½, ½), Ni on 1*a* (0, 0, 0) and O on 3*d* (½, 0, 0). The irreps are labeled using the **k** vector designation in the first BZ of the cubic unit cell, *i.e.*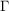, *X*, *M* and *R*. Since Ni is at the origin of the aristotype unit cell, we shifted the atomic coordinates in both monoclinic and orthorhombic lattices to have at least one Ni at the origin. Prior to the group-theory calculations, both monoclinic and orthorhombic lattices were converted to their respective standard sets, 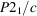 and *Pnma*, respectively, using the *CIF2Standard* tool (Kroumova *et al.*, 2003 ▸). The decomposition to represent the low-symmetry phases using the irreps of the supergroup *G* (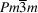) space group leads to the Wyckoff site splitting in Table 2 ▸. The symmetry-mode amplitudes are listed in Table 3 ▸. On the basis of such a splitting description, the number and symmetry vectors of the distortion modes can be calculated for each distorted phase.

**Figure 2:**
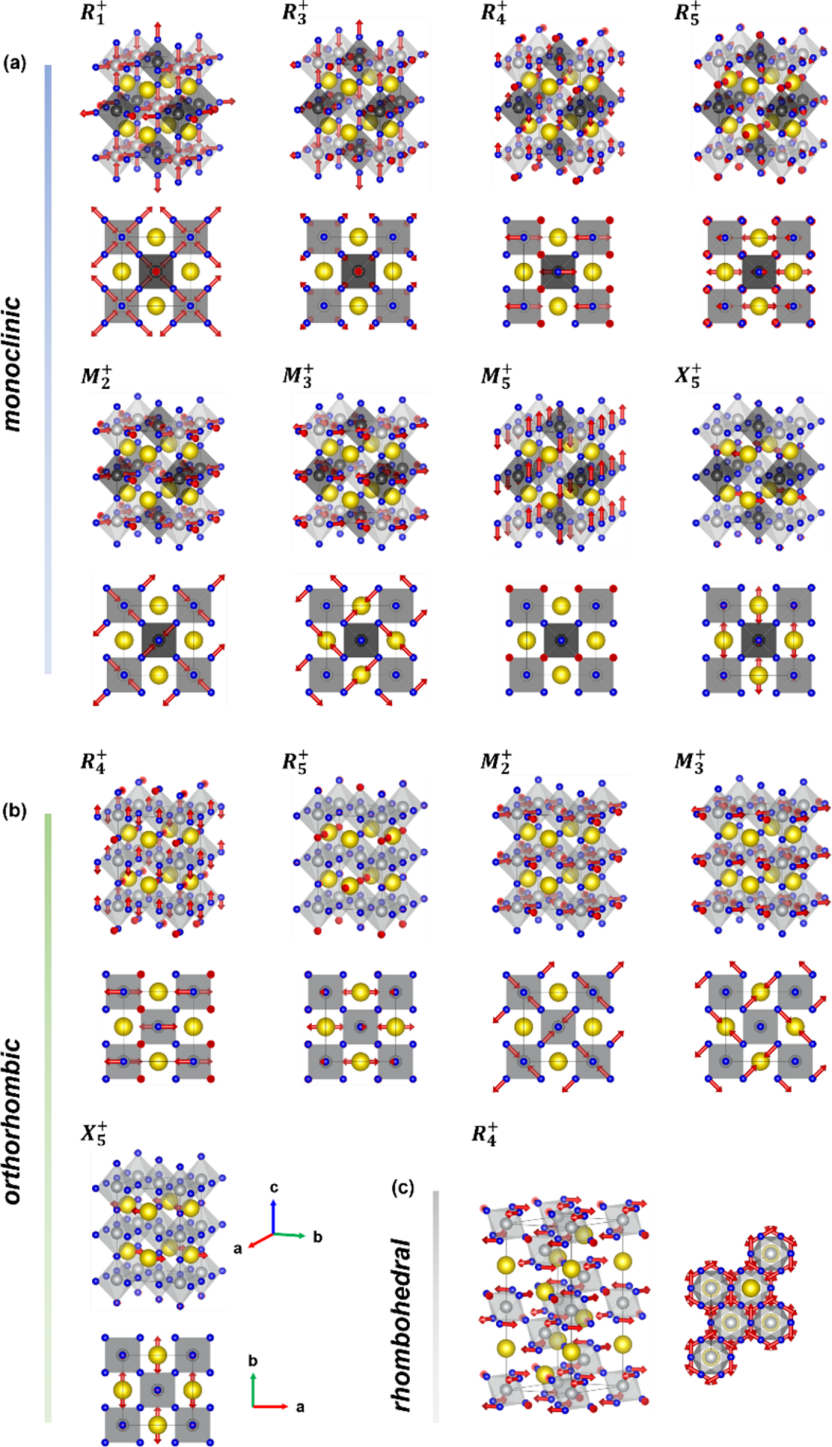
Schematic representation of the atomic displacements (polarization vectors) of symmetry-adapted modes for describing the symmetry-lowering transitions (*a*) 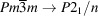, (*b*) 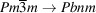 and (*c*) 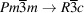. Pr, Ni and O atoms are drawn as yellow, gray and blue spheres, respectively, while the red arrows denote the amplitudes of the symmetry-adapted modes. To represent the two distinct nickel sites in the monoclinic phase, the octahedral units are colored with two different gray shades (Ni1^3+δ^, light; Ni2^3−δ′^, dark).

We started the symmetry-mode analysis from the rhombohedral phase, for which the static displacements for the aristo­type symmetry lowering come from one frozen mode 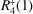 along the direction [111], wavevector **k** = (½, ½, ½), of the pseudo-cubic unit cell. It results from an out-of-phase octahedral [NiO_6_] tilt with Glazer’s symbol (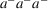) [see the mode representation in Fig. 2 ▸(*c*)]. The irrep 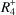 shows precisely the out-of-phase tilt along the *c* direction of the hexagonal cell, *i.e.* parallel to the pseudo-cubic direction [111]. The tilts lead to trigonal distortion of the rhombohedral phase and an additional degree of freedom for the oxygen fractional coordinate on 18*e* sites (*x*, 0, ¼) compared with the aristotype unit cell (Table 2 ▸).

In the orthorhombic phase, more degrees of freedom are enabled due to the Wyckoff site splitting resulting from seven distortion modes, as represented by five irreps, namely 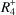, 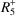, 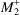, 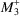 and 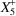 in Table 2 ▸. Pr site 1*b* enables two distortion modes [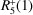 + 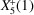] on Pr site 4*c* within the orthorhombic lattice. Their respective polarization vectors are mainly described by Pr translations along the *a* and *b* axes, respectively [Fig. 2 ▸(*b*)]. The oxygen 3*d* site splitting leads to five modes [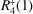 + 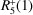 + 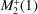 + 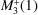 + 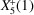] on O sites 4*c* and 8*d* of the *Pbnm* phase. From the symmetry-mode amplitudes in Table 3 ▸, we note that the orthorhombic distortion is mainly induced by the modes 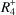 (0.529 Å) and 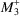 (0.334 Å) which describe the out-of-phase (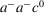) and in-phase (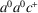) rotations of neighboring octahedra, respectively, to account for the Glazer symbol 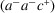 of the orthorhombic lattice. The polarization vectors of the distortion mode 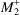 mimic an asymmetric octahedral stretching confined to the *ab* plane but exhibiting a low amplitude of 0.013 Å, and therefore having less impact on the global orthorhombic distortion and showing the minor role of any possible orbital ordering (Zhou & Goodenough, 2004 ▸; Mazin *et al.*, 2007 ▸).

In the monoclinic phase, 12 distortion modes as written by eight irreps, namely 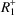, 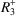, 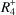, 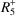, 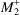, 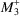, 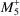 and 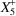 in Table 2 ▸, are allowed. In addition to the irreps from the orthorhombic distortion, four more [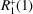, 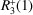, 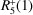 and 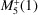] are needed to account for the charge disproportionation and, consequently, the charge ordering at long range of the monoclinic phase. In Fig. 2 ▸(*a*), we see that the 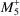 mode is an additional rotation of adjacent octahedra that accounts for the Glazer symbol 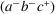 of the monoclinic unit cell. The polarization vectors of 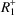 and 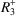 describe symmetric and asymmetric octahedral stretching modes with quite similar amplitudes of 0.062 and 0.042 Å, respectively, meaning that the charge disproportion below 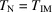 may be driven by oxygen stretching (breathing) modes. The asymmetric stretching is also known as Jahn–Teller-type mode because it distorts the octahedral cage to induce apical and basal oxygen splitting. Consequently, the charge transfer 2Ni^3+^ → Ni1^3+δ^ + Ni2^3−δ^ can be thought of as a phonon-mediated process, the oxygen stretching modes being essential to its progress.

**Figure 3:**
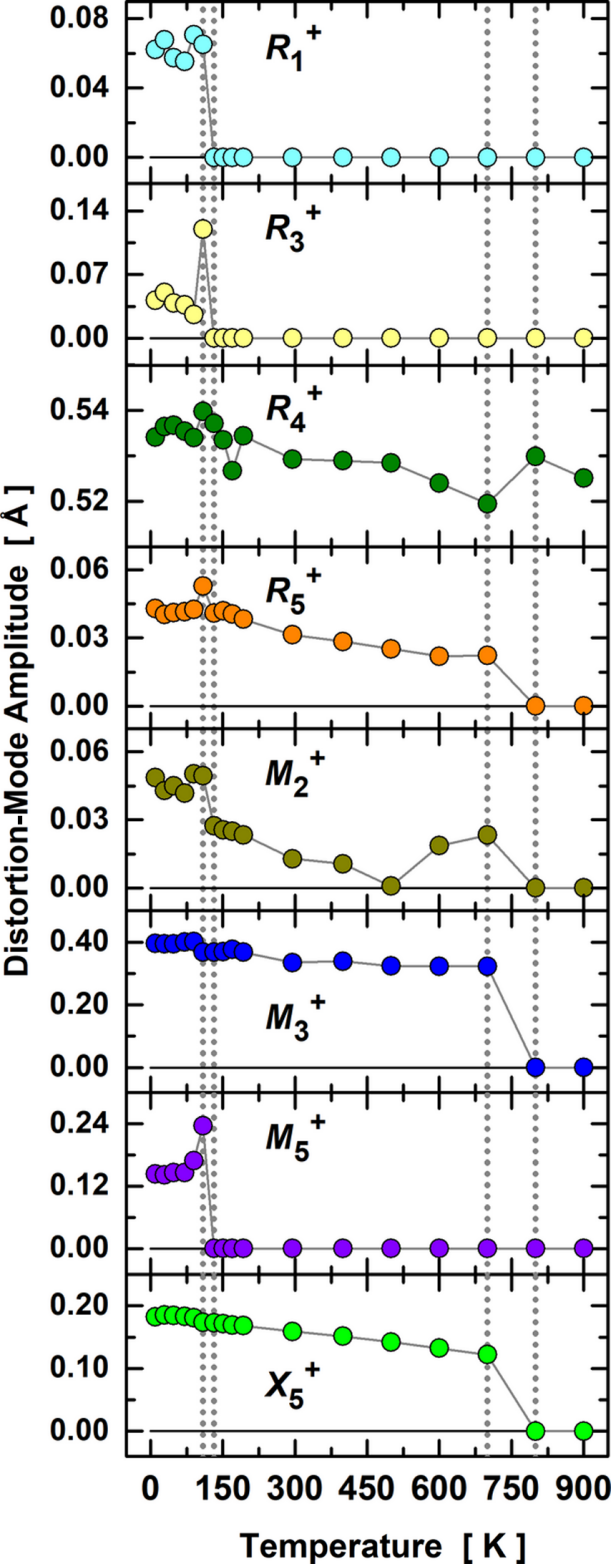
Temperature dependence of the symmetry-mode amplitudes across the transitions 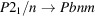 and 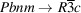. The amplitudes were normalized within the primitive unit cell of the aristotype high-symmetry structure. The dashed vertical lines delimit the onset ranges of the displacive structural transitions.

Using the temperature-dependent crystal structure of PrNiO_3_ in the broad range 10–900 K, we have analyzed the evolution of the symmetry-mode amplitudes across the phase transitions in the temperature range 10–900 K, as plotted in Fig. 3 ▸. The amplitudes are normalized with respect to the primitive unit cell of the aristotype cubic cell. All the mode amplitudes vanish for temperatures above 700 K except for 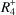, which is the sole mode for describing the rhombohedral distortion along the pseudo-cubic direction [111]. Below 700 K, the highest-amplitude modes 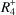 and 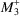 show a nearly temperature-independent behavior, with slight anomalies at 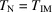 possibly induced by the 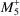 mode which also rotates the adjacent octahedral layers toward the monoclinic distortion. The mode amplitudes encompassing Pr atoms [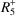 and 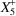] enhance the distortion when the temperature decreases and 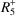 has a subtle change near 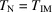. Interesting trends can be observed for the stretching modes 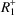, 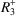 and 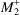, in view of their pronounced jumps at the onset of the insulator–metal transition.

In our previous work (Rodrigues *et al.*, 2023 ▸), we found strong evidence for spin–phonon coupling below 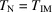 in PrNiO_3_ using X-ray absorption spectroscopy. The Debye–Waller exponent of the Ni—O pair bond indicated a softening behavior below the IM transition, attributed to the coupling of the Ni magnetic lattice and the stretching vibrational modes of the nickel octahedra. On the basis of the analysis presented here, we can now say that charge disproportionation is mediated by oxygen stretching modes, a hypothesis that was also raised recently (Gawryluk *et al.*, 2019 ▸). Our results demonstrate that both charge transfer and magnetic ordering are intimately entangled with phonon-mediated processes.

## 4. Conclusions

Our study presents a full symmetry-mode decomposition analysis of PrNiO_3_ across its temperature-induced structural phase transitions, enabled by high-quality and high-resolution SXRD data collected between 10 and 900 K. We identify oxygen stretching and Jahn–Teller-like modes as the primary drivers of charge disproportionation at ∼130 K (metal–insulator transition) and show that these distortions persist and evolve through the high-temperature orthorhombic to rhombo­hedral transition. The transition to the 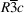 phase is characterized by the disappearance of all modes except a single octahedral [NiO_6_] tilt.

These results establish that phonon-mediated lattice distortions not only drive electronic and magnetic transitions but also govern structural evolution in both insulating and metallic states, offering a unified framework to understand phase competition in electron-correlated nickelates.

## Supplementary Material

Additional tables with structural parameters. DOI: 10.1107/S1600576725009380/hat5010sup1.pdf

## Figures and Tables

**Table 1:** Summary of reported transition temperatures (K) of PrNiO_3_ nickelate Abbreviations: SXRD synchrotron X-ray diffraction, XANES X-ray absorption near-edge structure, EXAFS extended X-ray absorption fine structure, XRD X-ray diffraction and NPD neutron powder diffraction.

Experiment	*T* range (K)	*P*2_1_/*n* → *Pbnm*	*Pbnm* → 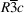	Technique
Rodrigues *et al.* (2024 ▸)	10–900	130	750	SXRD, XANES
Rodrigues *et al.* (2023 ▸)	10–300	125		EXAFS
Gawryluk *et al.* (2019 ▸)	1.5–300	130		SXRD, NPD
Huang *et al.* (1990 ▸)	473–873		773	XRD
García-Muñoz *et al.* (1992 ▸)	1.5–293	135		NPD
Acosta-Alejandro *et al.* (2008 ▸)	105–136	128–136		XANES
Medarde *et al.* (2008 ▸)	10–170	130		NPD
Medarde *et al.* (1992 ▸)	77–295	135		XANES

**Table 2:** Symmetry-mode analysis of the PrNiO_3_ crystal structure elucidating the Wyckoff site splitting for each low-symmetry phase and the symmetry-adapted modes responsible for the symmetry lowering induced under increasing temperature

Supergroup *G*	Subgroup *H*	Symmetry-adapted modes
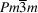 (No. 221)	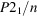 (No. 14)	
Pr 1*b*	Pr 4*e*	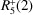 + 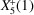
Ni 1*a*	Ni1 2*d*, Ni2 2*c*	
O 3*d*	O1 4*e*, O2 4*e*, O3 4*e*	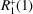 + 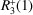 + 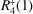 + 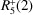 + 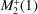 + 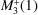 + 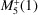 + 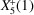

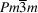 (No. 221)	*Pbnm* (No. 62)	
Pr 1*b*	Pr 4*c*	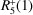 + 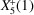
Ni 1*a*	Ni 4*b*	
O 3*d*	O1 4*c*, O2 8*d*	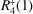 + 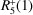 + 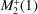 + 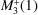 + 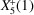

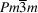 (No. 221)	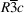 (No. 167)	
Pr 1*b*	Pr 6*a*	
Ni 1*a*	Ni 6*b*	
O 3*d*	O 18*e*	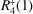

**Table 3:** Amplitudes of the symmetry-adapted modes as normalized within the primitive unit cell of the aristotype high-symmetry structure, together with their respective direction and dimension (multiplicity) for the monoclinic (10 K), orthorhombic (295 K) and rhombohedral (900 K) phases

Irreps	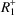	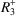	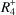	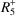	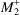	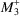	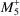	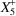
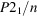 (No. 14), *T* = 10 K								
Direction	(*a*)	(*a* 0)	(0 *aa*)	(−*ba* −*a*)	(*a* 0 0)	(*a* 0 0)	(*aa* 0 0 *a* −*a*)	(0 0 0 −*a* 0 0)
Dimension	1	1	1	4	1	1	1	2
Amplitude (Å)	0.062	0.042	0.534	0.043	0.048	0.396	0.143	0.182

*Pbnm* (No. 62), *T* = 295 K								
Direction			(0 *aa*)	(0 *a* −*a*)	(*a* 0 0)	(*a* 0 0)		(0 0 0 −*a* 0 0)
Dimension			1	2	1	1		2
Amplitude (Å)			0.529	0.031	0.013	0.334		0.159

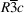 (No. 167), *T* = 900 K								
Direction			(*aaa*)					
Dimension			1					
Amplitude (Å)			0.525					
