# Supplementary material for: Revisiting the structures and phase transitions of PrNiO3 nickelate using symmetry-mode analysis
Source: J Appl Crystallogr. 2025 Nov 26;58(Pt 6):2112–8. doi: 10.1107/S1600576725009380 (PMC12810527; doi:10.1107/S1600576725009380)
Supplement: Supplementary file 1 [file j-58-02112-sup1.pdf]

JOURNAL OF
APPLIED
CRYSTALLOGRAPHY

Volume 58 (2025)

Supporting information for article:

Revisiting the structures and phase transitions of PrNiO3 nickelate using symmetry-mode analysis

Wajdi Cherif, José Antonio Alonso and João Elias F. S. Rodrigues

Table S1. Structural parameters of the $PrNiO_3$ nickelate with the monoclinic phase ($P2_1/n$).

	**10 K**	**29 K**	**48 K**	**71 K**	**90 K**	**109 K**
a (Å)	5.41224(2)	5.41169(2)	5.41134(2)	5.41123(2)	5.41138(2)	5.40913(2)
b (Å)	5.38484(2)	5.38498(1)	5.38474(2)	5.38428(2)	5.38370(2)	5.37660(2)
c (Å)	7.61503(2)	7.61696(2)	7.61873(2)	7.62029(2)	7.62163(2)	7.61400(3)
β (°)	90.06638(17)	90.06580(16)	90.06450(17)	90.06299(16)	90.06148(19)	90.00424(181)
V ($Å^3$)	221.933(1)	221.972(1)	221.999(1)	222.021(1)	222.042(1)	221.436(1)
ρ ($g.cm^{-3}$)	7.411	7.409	7.408	7.408	7.407	7.427
**Pr at 4*e* (*x*, *y*, *z*)**						
x	0.99385(7)	0.99352(7)	0.99376(7)	0.99380(7)	0.99377(8)	0.99425(10)
y	0.03297(4)	0.03308(4)	0.03302(4)	0.03285(4)	0.03237(4)	0.03122(5)
z	0.24947(11)	0.24977(11)	0.24964(11)	0.24931(11)	0.25017(14)	0.25157(18)
Pr B_{iso} ($Å^2$)	0.282(4)	0.316(4)	0.343(4)	0.357(4)	0.390(4)	0.351(6)
**O1 at 4*e* (*x*, *y*, *z*)**						
x	0.07144(65)	0.07177(65)	0.07154(68)	0.07230(69)	0.07304(77)	0.07586(91)
y	0.49210(60)	0.49087(61)	0.49107(62)	0.49107(62)	0.49105(68)	0.49229(82)
z	0.25920(82)	0.26080(76)	0.25851(89)	0.25813(92)	0.25814(107)	0.26777(152)
O1 B_{iso} ($Å^2$)	0.338(39)	0.609(76)	0.635(78)	0.741(80)	0.734(53)	0.767(47)
**O2 at 4*e* (*x*, *y*, *z*)**						
x	0.71759(106)	0.71655(102)	0.71735(104)	0.71622(104)	0.71463(111)	0.72068(136)
y	0.28648(117)	0.28650(115)	0.28642(117)	0.28705(115)	0.28677(120)	0.29142(154)
z	0.04761(71)	0.04764(70)	0.04820(71)	0.04765(71)	0.04925(75)	0.05479(85)
O2 B_{iso} ($Å^2$)	0.338(39)	0.416(38)	0.188(50)	0.160(49)	0.295(42)	0.236(54)
**O3 at 4*e* (*x*, *y*, *z*)**						
x	0.21792(111)	0.21833(108)	0.21789(110)	0.21734(109)	0.22024(118)	0.22032(147)
y	0.20393(121)	0.20549(121)	0.20500(123)	0.20513(121)	0.20302(128)	0.21411(166)
z	0.97890(76)	0.97866(74)	0.97890(75)	0.97945(73)	0.98216(76)	0.98902(95)
O3 B_{iso} ($Å^2$)	0.338(39)	0.416(38)	0.188(50)	0.160(49)	0.176(45)	0.198(61)
**Ni1 at 2*d* (1/2, 0, 0)**						
Ni1 B_{iso} ($Å^2$)	0.155(38)	0.363(39)	0.317(41)	0.297(42)	0.353(42)	0.333(55)
<Ni1–O> (Å)	1.9121(25)	1.9092(25)	1.9151(26)	1.9168(26)	1.9089(29)	1.9106(38)
Ni1 valence	3.279(22)	3.306(22)	3.250(23)	3.233(23)	3.306(26)	3.348(37)
Ni1 $\Delta_d \times 10^{-4}$	5.339	5.455	4.698	4.171	4.885	17.962
**Ni2 at 2*c* (1/2, 0, 1/2)**						
Ni2 B_{iso} ($Å^2$)	0.305(40)	0.187(36)	0.127(37)	0.185(43)	0.171(38)	0.186(52)
<Ni2–O> (Å)	1.9827(25)	1.9859(25)	1.9804(26)	1.9794(26)	1.9888(29)	1.9848(38)

Ni2 valence	2.698(18)	2.675(18)	2.713(19)	2.720(19)	2.651(21)	2.719(27)
Ni2 $\Delta_d \times 10^{-4}$	1.823	2.108	1.456	1.285	1.006	11.743
**Charge disproportionation**						
$\delta_{eff} = (\delta + \delta')/2$	0.290	0.315	0.268	0.256	0.327	0.314
**Reliability factors**						
R_p (%)	9.60	9.28	9.35	9.32	10.3	15.0
R_{wp} (%)	14.9	14.5	14.8	14.6	15.8	19.5
R_{exp} (%)	3.83	3.87	3.96	4.01	4.06	4.03
R_{Bragg} (%)	5.06	4.80	5.13	4.96	5.14	7.17
R_f (%)	6.59	6.37	7.36	7.22	6.94	7.62

Table S2. Structural parameters of the $PrNiO_3$ nickelate with the orthorhombic phase ($Pbnm$).

	**132 K**	**151 K**	**170 K**	**193 K**	**295 K**	**400 K**	**500 K**	**600 K**	**700 K**
a (Å)	5.41042(1)	5.41114(1)	5.41197(1)	5.41259(1)	5.41846(1)	5.42547(1)	5.43304(1)	5.44060(1)	5.44815(1)
b (Å)	5.37702(1)	5.37720(1)	5.37748(1)	5.37754(1)	5.38020(1)	5.38406(1)	5.38863(1)	5.39333(1)	5.39813(1)
c (Å)	7.61604(1)	7.61731(2)	7.61856(1)	7.61938(2)	7.62606(1)	7.63336(1)	7.64101(1)	7.64840(1)	7.65552(1)
V ($Å^3$)	221.565(1)	221.639(1)	221.721(1)	221.773(1)	222.318(1)	222.979(1)	223.703(1)	224.427(1)	225.148(1)
ρ ($g.cm^{-3}$)	7.423	7.421	7.418	7.416	7.398	7.376	7.352	7.328	7.305
**Pr at 4*c* (*x*, *y*, 1/4)**									
x	0.99420(7)	0.99408(7)	0.99469(8)	0.99429(7)	0.99476(6)	0.99502(7)	0.99536(7)	0.99595(7)	0.99635(8)
y	0.03065(4)	0.03041(4)	0.03013(4)	0.02997(4)	0.02860(3)	0.02713(3)	0.02549(3)	0.02386(3)	0.02205(3)
Pr B_{iso} ($Å^2$)	0.393(4)	0.408(4)	0.440(4)	0.452(4)	0.585(3)	0.718(4)	0.867(4)	1.013(4)	1.179(5)
**O1 at 4*c* (*x*, *y*, 1/4)**									
x	0.07392(66)	0.07356(62)	0.07285(66)	0.07308(66)	0.07121(49)	0.07060(50)	0.06972(49)	0.06847(46)	0.06681(48)
y	0.49073(63)	0.49065(60)	0.49120(63)	0.49135(63)	0.49256(48)	0.49276(50)	0.49308(50)	0.49426(48)	0.49490(52)
O1 B_{iso} ($Å^2$)	0.805(71)	0.846(67)	0.905(73)	0.866(71)	0.981(55)	1.131(59)	1.281(60)	1.273(57)	1.300(62)
**O2 at 8*d* (*x*, *y*, *z*)**									
x	0.71836(49)	0.71814(46)	0.71734(48)	0.71811(49)	0.72019(39)	0.71956(41)	0.71990(42)	0.71830(41)	0.71787(45)
y	0.28670(53)	0.28659(48)	0.28728(51)	0.28622(52)	0.28219(40)	0.28242(41)	0.27995(41)	0.27825(41)	0.27782(45)
z	0.03351(33)	0.03322(31)	0.03268(33)	0.03357(33)	0.03384(25)	0.03410(26)	0.03448(26)	0.03452(25)	0.03475(27)
O2 B_{iso} ($Å^2$)	0.208(40)	0.158(37)	0.237(40)	0.257(41)	0.490(33)	0.593(36)	0.766(38)	0.945(39)	1.109(44)
**Ni at 4*b* (1/2, 0, 0)**									
Ni B_{iso} ($Å^2$)	0.240(7)	0.256(6)	0.268(7)	0.254(7)	0.309(5)	0.395(6)	0.491(6)	0.584(6)	0.680(7)
<Ni–O> (Å)	1.9431(9)	1.9431(9)	1.9432(9)	1.9434(9)	1.9424(7)	1.9445(7)	1.9457(7)	1.9474(7)	1.9490(9)
Ni valence	2.997(7)	2.997(7)	2.996(7)	2.994(7)	3.001(6)	2.984(6)	2.974(6)	2.962(6)	2.950(7)
Ni $\Delta_d \times 10^{-4}$	0.556	0.465	0.435	0.378	0.110	0.063	0.018	0.386	0.589

**Reliability factors**									
R_p (%)	8.90	8.52	8.79	8.81	7.02	7.53	7.51	7.57	8.28
R_{wp} (%)	14.5	13.6	14.4	14.4	11.1	11.5	11.3	10.9	11.9
R_{exp} (%)	3.91	3.99	4.10	4.15	2.03	2.99	2.45	2.63	2.64
R_{Bragg} (%)	4.61	4.33	4.59	4.62	3.39	3.75	3.65	3.48	4.12
R_f (%)	6.39	5.97	6.46	6.48	5.34	5.99	6.18	6.01	6.92

Table S3. Structural parameters of the $PrNiO_3$ nickelate with the rhombohedral phase ($R\bar{3}c$).

	**800 K**	**900 K**
a (Å)	5.45984(1)	5.46373(1)
c (Å)	13.09255(1)	13.11978(2)
V (Å^3)	337.998(1)	339.184(1)
ρ (g.cm^{-3})	7.299	7.273
**Pr at 6*a* (0, 0, 1/4)**		
Pr B_{iso} (Å^2)	1.343(5)	1.489(5)
**O1 at 18*e* (*x*, 0, 1/4)**		
x	0.44324(28)	0.44374(28)
O1 B_{iso} (Å^2)	1.601(32)	1.817(33)
**Ni at 6*b* (0, 0, 0)**		
Ni B_{iso} (Å^2)	0.749(7)	0.808(7)
<Ni–O> (Å)	1.9418(4)	1.9436(4)
Ni valence	3.005(3)	2.991(3)
Ni $\Delta_d \times 10^{-4}$	-0.002	0.001
**Reliability factors**		
R_p (%)	9.40	8.92
R_{wp} (%)	12.3	11.7
R_{exp} (%)	2.50	2.60
Bragg R-factor	2.94	2.78
Rf-factor	5.78	6.66
